# Effector‐mediated discovery of a novel resistance gene against *Bremia lactucae* in a nonhost lettuce species

**DOI:** 10.1111/nph.14741

**Published:** 2017-08-21

**Authors:** Anne K. J. Giesbers, Alexandra J. E. Pelgrom, Richard G. F. Visser, Rients E. Niks, Guido Van den Ackerveken, Marieke J. W. Jeuken

**Affiliations:** ^1^ Laboratory of Plant Breeding Wageningen University & Research 6700 AJ Wageningen the Netherlands; ^2^ Plant–Microbe Interactions Department of Biology Utrecht University 3584 CH Utrecht the Netherlands

**Keywords:** avirulence, effector‐triggered immunity (ETI), *Lactuca saligna*, lettuce downy mildew, nonhost resistance, oomycete, quantitative trait locus (QTL), *R* gene

## Abstract

- Candidate effectors from lettuce downy mildew (*Bremia lactucae*) enable high‐throughput germplasm screening for the presence of resistance (*R*) genes. The nonhost species *Lactuca saligna* comprises a source of *B. lactucae R* genes that has hardly been exploited in lettuce breeding. Its cross‐compatibility with the host species *L. sativa* enables the study of inheritance of nonhost resistance (NHR).
- We performed transient expression of candidate RXLR effector genes from *B. lactucae* in a diverse *Lactuca* germplasm set. Responses to two candidate effectors (BLR31 and BLN08) were genetically mapped and tested for co‐segregation with disease resistance.
- BLN08 induced a hypersensitive response (HR) in 55% of the *L. saligna* accessions, but responsiveness did not co‐segregate with resistance to Bl:24. BLR31 triggered an HR in 5% of the *L. saligna* accessions, and revealed a novel R gene providing complete *B. lactucae* race Bl:24 resistance. Resistant hybrid plants that were BLR31 nonresponsive indicated other unlinked *R* genes and/or nonhost QTLs.
- We have identified a candidate avirulence effector of *B. lactucae* (BLR31) and its cognate *R* gene in *L. saligna*. Concurrently, our results suggest that *R* genes are not required for NHR of *L. saligna*.

## Introduction

Nonhost resistance (NHR) is defined as immunity occurring in all genotypes of a plant species against all genotypes of a specific pathogen (Heath, 1981; Niks, 1987; Niks & Marcel, 2009). Understanding the mechanisms of NHR may lead to the development of durable and broad‐spectrum disease resistance in crop plants.

If a pathogen breaks through a plant's preformed defences, it can be recognized by that plant through two overlapping layers of immunity (Jones & Dangl, 2006). The first layer depends on the recognition of pathogen‐derived molecules, also called pathogen‐associated molecular patterns (PAMPs). PAMP recognition, commonly through pattern recognition receptors, can lead to resistance and is referred to as PAMP‐triggered immunity (PTI). To counteract PTI, pathogens secrete effector molecules targeting host intracellular compartments that enhance infection through the manipulation of host cellular processes, leading to effector‐triggered susceptibility (ETS).

The second layer of immunity is triggered when host cells recognize avirulence effectors through resistance (R) proteins. In oomycetes, such as *Phytophthora* and downy mildew, effectors are often characterized by RXLR and EER amino acid motifs (Rouxel & Balesdent, 2010; Anderson *et al*., 2015). These conserved amino acid motifs are thought to mediate the entry of oomycete effectors into host cells (Whisson *et al*., 2007). R proteins are typically nucleotide‐binding leucine‐rich repeat (NLR) proteins that act on their own or in pairs to recognize effectors directly, or indirectly by the detection of manipulation of a plant target (Van Der Biezen & Jones, 1998; Wu *et al*., 2016). Recognition of effectors leads to effector‐triggered immunity (ETI) and is usually associated with a hypersensitive response (HR) resulting in localized cell death.

The application of (candidate) effectors to host plant leaf tissue through transient expression triggered HRs that are associated with novel and already known *R* genes in some pathosystems (Vleeshouwers *et al*., 2008; Rietman *et al*., 2012; Gascuel *et al*., 2016; Lenman *et al*., 2016). Recognition of effectors in nonhost species is often assumed to contribute to NHR (Wroblewski *et al*., 2009; Schulze‐Lefert & Panstruga, 2011; Lee *et al*., 2014; Adlung *et al*., 2016). As NHR, by definition, implies that a complete species is resistant, it should be studied at the species level.

Only a few screenings of transiently expressed effectors in multiple accessions of nonhost species have been described, namely *Pseudomonas* and *Ralstonia* effectors in 19 *Lactuca sativa* accessions (Wroblewski *et al*., 2009), *B. lactucae* effectors in 52 *Lactuca saligna* accessions (Stassen *et al*., 2013), *Phytophthora infestans* effectors in 42 *Capsicum annuum* accessions (Lee *et al*., 2014) and *Xanthomonas campestris* pv *vesicatoria* effectors in 46 *Nicotiana tabacum* accessions (Adlung *et al*., 2016). Effector‐induced response patterns in these studies can be divided into responses induced in a broad range of accessions and responses induced in a narrow range of accessions. In all of these studies, some effectors triggered an HR in a broad range of accessions from the nonhost species, ranging from 52% (Lee *et al*., 2014) to 100% (Wroblewski *et al*., 2009; Adlung *et al*., 2016) responsive accessions.

Yet, it is still unclear to what extent NHR is a result of ETI. A way out could be to cross the resistant nonhost species to a susceptible host, and to find co‐segregation between effector‐induced HR and resistance in the progeny. However, host and nonhost plants are usually sexually incompatible, which hampers classical genetic studies of NHR. The plant pathosystem of lettuce (*Lactuca* spp.) and downy mildew (caused by the oomycete *Bremia lactucae* (Michelmore & Wong, 2008)) provides a rare opportunity to study the inheritance of NHR. The cultivated host species *L. sativa* is cross‐compatible with the nonhost species *L. saligna* (both self‐pollinating, diploid species). Since 1976, several studies have indicated that *L. saligna* accessions are totally disease free (Netzer *et al*., 1976; Norwood *et al*., 1981; Lebeda, 1986; Gustafsson, 1989; Bonnier *et al*., 1992; Petrželová *et al*., 2011; Van Treuren *et al*., 2011). Histological studies have suggested that germinated conidia in *L. saligna* are arrested before normal hyphae formation (Lebeda *et al*., 2008; Zhang *et al*., 2009b).

Based on our previous genetic studies, the NHR of *L. saligna* CGN05271 seems to be explained by multiple race‐nonspecific quantitative effects (quantitative trait loci, QTLs), although the QTL × QTL interactions have not yet been solved (Jeuken & Lindhout, 2002; Jeuken *et al*., 2008; Zhang *et al*., 2009a; Den Boer *et al*., 2014). In addition, a few *L. saligna* accessions are known to contain dominant monogenic race‐specific *R* genes to *B. lactucae* (Parra *et al*., 2016). The race‐specific effect of these *R* genes makes it unlikely that they are the main factor explaining NHR.

Since the 1970s, more than 50 *R* genes have been deployed in lettuce breeding (Parra *et al*., 2016). Most of them originated from the primary gene pool species *L. sativa* or *L. serriola* (Parra *et al*., 2016). These genes do not provide broad‐spectrum resistance and are rapidly broken by *B. lactucae*. At least 25 of these *R* genes co‐localize to one of the three major clusters of candidate *R* genes (NLRs) in the lettuce genome (Christopoulou *et al*., 2015). The resistance of secondary gene pool species, including the nonhost *L. saligna*, to *B. lactucae* is relatively uncharacterized and unexploited, because of a low success rate of crossings with cultivated lettuce and reduced fertility of the F_1_ generation. The testing of computationally predicted effector genes (referred to as candidate effectors) in the nonhost *L. saligna* could be a helpful tool to preselect accessions with an HR and therefore potential *R* genes. By only using these preselected accessions for further mapping studies, a lot of time and effort involved in the development of wide crosses and segregating populations could be saved. In this manner, potential sources of *R* genes may be expanded beyond the primary gene pool in an efficient way.

Compared with classical disease tests, the screening of *Lactuca* germplasm with effectors by transient expression assays could be more rapid and effective for the identification of new *R* genes. Furthermore, *R* gene repertoires can be compared between accessions. In an effector transient expression assay, the effect of a single effector is observed, whereas a classical disease test with spores reflects the sum of effects from a mix of effectors. In classical disease tests, the resistance of a plant may be the result of the activation of various *R* genes, triggered by separate effectors that are masking each other's effects. As a result, potential resistances may remain hidden (reviewed in Vleeshouwers *et al*., 2011). Effectoromics has shown its potential in the potato–*Phytophthora* pathosystem by the identification of new *R* genes, accelerated *R* gene cloning, detection of resistance specificities and *R* gene deployment in agriculture (Vleeshouwers & Oliver, 2014). Possibly, this success can be transferred to the lettuce–downy mildew pathosystem. Lettuce is definitely in need of new *R* genes as *B. lactucae* variability rapidly defeats the latest introduced *R* genes.

Previously, responses to two *B. lactucae* Bl:24 effectors have been mapped in *Lactuca* germplasm (Stassen *et al*., 2013). Effector BLG03 triggered a response specifically in *L. sativa* cultivars containing the *Dm2 R* gene, whereas effector BLG01 induced a response in the majority (90%) of tested *L. saligna* accessions. Both responses were not associated with disease resistance against the *B. lactucae* race from which the effector was derived.

In the current study, we tested some new candidate effectors with a specific focus on the nonhost *L. saligna*. The objectives were to obtain a broader picture of the responsiveness of *L. saligna* to *B. lactucae* effectors, and to verify whether responsiveness to effectors in this nonhost contributes to disease resistance. We have identified two new candidate effectors that induce a response in the nonhost *L. saligna*. Responsiveness to only one candidate effector co‐segregated with disease resistance.

## Materials and Methods

### Plant materials

*Lactuca* L. germplasm (*n *=* *150) was selected to include a wide range of *Bremia lactucae* resistances (*n *=* *53), including a differential set of *L. sativa* cultivars with single dominant *R* genes, and a broad diversity of accessions from the primary (*n *=* *72: *L. sativa*,* L. serriola*,* L. aculeata*,* L. altaica*) and secondary (*n *=* *78: *L. saligna*,* L. virosa*,* L. georgica*) gene pool of lettuce (Supporting Information Table S1a).

### *Lactuca saligna* phenogram

A phenogram of 45 *L. saligna* accessions was based on 423 aflp‐fragments, derived from eight primer combinations. Distances were calculated using the ‘dist’ function in the R package ‘stats’ (R Core Team, 2016). A tree was obtained using the neighbour‐joining method in the R package ‘ape’ (Paradis *et al*., 2004). Two *L. sativa* cultivars represented the outgroup.

### Mapping populations

The responsiveness to candidate effectors and *R* genes against *B. lactucae* was mapped in segregating populations, such as F_2_, backcross (BC1) or a previously developed set of backcross inbred lines (BILs). F_1_ plants from *L. saligna* accessions × *L. sativa* cv Olof were retrieved with difficulty, with variable success rates and with a severely reduced (although still sufficient) fertility for further inbreeding (F_2_) and backcrossing (BC1). F_1_s were used as a mother in a backcross to *L. sativa* cv Olof to obtain three distinct BC1 populations, or selfed to obtain an F_2_ population (see later Fig. 2b). Populations were named after their generation (BC1 or F_2_) and the CGN number of the parental *L. saligna* accession, resulting in the following population names: BC1_CGN05947, BC1_CGN05304, BC1_CGN05318 and F2_CGN05310. The set of 29 BILs with *L. saligna* CGN05271 introgression segments in a *L. sativa* cv Olof background (Jeuken & Lindhout, 2004) are referred to as BILs_CGN05271.

### DNA isolation and genotyping

DNA was isolated from plant leaf tissue by either a high‐throughput NaOH method (Wang *et al*., 1993) or a cetyltrimethylammonium bromide (CTAB) method (Jeuken *et al*., 2001). Polymorphisms between PCR products of *L. saligna* and *L. sativa* alleles were visualized by high‐resolution melting curve differences on a LightScanner System (Den Boer *et al*., 2014). Populations were genotyped to map their effector response and/or major *R* gene(s). Genetic markers were analysed at the major resistance cluster (MRC) regions in *L. sativa* (Christopoulou *et al*., 2015), if necessary followed by markers in the rest of the genome (primer sequences, Table S2). Genetic map distances are derived from a reference F_2_ genetic linkage map of cross *L. saligna* CGN05271 × *L. sativa* cv Olof (Jeuken *et al*., 2001). Physical map locations refer to the *L. sativa* cv Salinas reference lettuce genome V8 (Reyes‐Chin‐Wo *et al*., 2017; https://lgr.genomecenter.ucdavis.edu/). Here, we use the chromosome numbering and orientation of the reference *L. sativa* physical map, in contrast with our previous publications. In order to relate previously reported trait locations to the mapped loci in the current study, Table S3 presents the correspondence between the chromosome numbering and orientation of the two maps. We use the following allele notation: w, wild lettuce (*L. saligna*) allele; c, cultivated lettuce (*L. sativa*) allele. Genotype notation: ww, homozygous *L. saligna*; wc, heterozygous; cc, homozygous *L. sativa*.

### Candidate effector identification and cloning

RNA was isolated from *B. lactucae* race Bl:24 spores and infected lettuce, as described by Stassen *et al*. (2012). Total RNA was further processed using the Illumina mRNA‐Seq sample preparation kit and sequenced (Illumina HiSeq2000). Total raw yields were 36 and 41 Mb for infected lettuce and *B. lactucae* spores, respectively, with ≥Q30 scores of 77.5% and 85.8%. The *B. lactucae* transcriptome was assembled using SOAPdenovo‐Trans release 1.03 (Luo *et al*., 2012) using default settings with the following adjustments: avg_ins = 120; map_len = 32; max_rd_len = 100; and K = 21. Candidate coding regions within the transcript sequences were identified using TransDecoder (Haas *et al*., 2013). The resulting protein sequences were analysed with SignalP3.0 (Bendtsen *et al*., 2004) to construct a secretome. To identify effector candidates, proteins were screened for RXLR‐like, dEER and LXLFLAK motifs. *Bremia lactucae* effector candidates were cloned (primer sequences, Table S2), sequenced and electrotransformed into *Agrobacterium tumefaciens* C58C1 (pGV2260), as described by Stassen *et al*., 2013. Searches for best Blast hits were performed using BlastP on nonredundant GenBank CDS translations+PDB+SwissProt+PIR+PRF excluding environmental samples from WGS projects (https://blast.ncbi.nlm.nih.gov, accessed 16 March 2017).

### Quantitative polymerase chain reaction (qPCR)

Time course qPCR experiments were performed on 3–4‐d‐old *L. sativa* cv Olof seedlings in a similar manner to that described by Stassen *et al*. (2013). Cotyledons were collected at 3 h after infection, 1 d post‐inoculation (dpi), 3 dpi and 6 dpi. Expression levels were determined as the number of qPCR cycles required for the abundance of each amplicon to reach the cycle threshold (Ct) level. We performed three independent experiments, each with three biological replicates (two technical replicates each). *Bremia lactucae* actin expression levels were calculated as ΔCt values relative to *L. sativa* actin. Effector expression levels were calculated as ΔCt values relative to *B. lactucae* actin (primer sequences, Table S2).

### *Agrobacterium*‐mediated transient transformation

*Agrobacterium tumefaciens* strains with cloned candidate effectors were grown in selective media, resuspended to an OD_600_ of 0.5 and infiltrated into lettuce leaves according to Stassen *et al*. (2013). Strains containing a vector with yellow fluorescent protein (YFP) or a cell death‐inducing protein of *Phytophthora sojae* (PsojNIP) were included as negative and positive controls, respectively.

Per plant genotype, we infiltrated two leaves with Agrobacterium strains, but, for the F2_CGN05310 per plant, only one leaf was infiltrated. In the *Lactuca* germplasm screening, each leaf was infiltrated with candidate effectors, YFP and PsojNIP. In the mapping populations, each leaf was infiltrated with the candidate effector and YFP. PsojNIP was applied to all parental lines, and to one leaf in BC1_05947 and BC1_05318. For the set of 29 BILs, on average, two plants per BIL were infiltrated. In the germplasm screening, the majority of accessions were assessed in two or three independent experiments. Subsequently, responses in a subset of secondary gene pool accessions were verified (Table S1b).

Plants were infiltrated at an age of 28–42 d. At 5–8 dpi, we observed symptoms at the upper and lower leaf surfaces. Plant responses to candidate effectors, positive (PsojNIP) and negative (YFP) controls were scored on a scale from 0 to 3 in half unit steps according to the following classification: 0, no visible symptoms; 1, bleaching or yellowing (chlorosis); 2, strong yellowing or cell death; or 3, strong cell death. The negative control YFP consistently elicited a score between 0 and 1, which was considered as the background response to *Agrobacterium*. We corrected the plant score to candidate effectors by subtracting the plant score to YFP. Therefore, the ‘plant response to the effector’ per plant is defined as the difference between the score to the candidate effector and the score to YFP. An average plant response of ≥ 1 to candidate effectors in the germplasm screening was considered as a robust response. In the segregating populations, also lower responses, as low as 0.3, were qualified as a response to an effector on the basis of the co‐segregation with resistance and/or the responsible *L. saligna* allele.

### *Bremia lactucae* disease test

Per mapping population, independent detached leaf assays were conducted on 5–8‐wk‐old adult plants, as described previously in Jeuken & Lindhout (2002) with some modifications. Four leaf squares (2 × 2 cm^2^) from four fully extended leaves were collected per plant and inoculated with (2–4) × 10^5^ spores ml^−1^ of *B. lactucae* race Bl:24. The following control lines were included: parental *L. saligna* accessions (complete resistance), *L. sativa* cv Iceberg and combi‐BIL (4.1 + 6.3) + 8.2 (high levels of quantitative resistance; Zhang *et al*., 2009b; Den Boer *et al*., 2014) and *L. sativa* cv Olof and *L. sativa* cv Cobham Green (susceptible). The infection severity level (ISL) was scored visually as the percentage of leaf area covered with sporangiophores between 10 and 14 d after inoculation. Relative infection severity (RIS) levels were calculated as a percentage relative to ISL on the susceptible parent *L. sativa* cv Olof. Plants with RIS levels ≤ 10% were considered to be highly resistant.

## Results

### Two *B. lactucae* candidate effectors trigger an HR in *L. saligna* accessions

Sixteen candidate effectors with RXLR(‐like) motifs were predicted from newly generated *B. lactucae* transcriptome data (described in the Materials and Methods section) and could be added to the 34 published previously (Stassen *et al*., 2012). Responsiveness to these new candidate effectors was tested by *Agrobacterium*‐mediated transient expression on a *Lactuca* germplasm set (*n *=* *150). The tested lines were selected from the primary and secondary gene pools of cultivated lettuce (*L. sativa*), for a wide range of resistances to *B. lactucae*, and for a broad range of geographical origins. Plants showed a range of macroscopic responses to candidate effector application, from no reaction (0) to chlorosis (1) and little or severe cell death (2 and 3) (described in the Materials and Methods section). Here, we report on two candidate effectors, BLR31 and BLN08 (GenBank accession numbers KY940276 and KY940275), that induced a robust response (average ≥ 1) in the secondary gene pool species *L. saligna* (Table S1).

**Figure 1:**
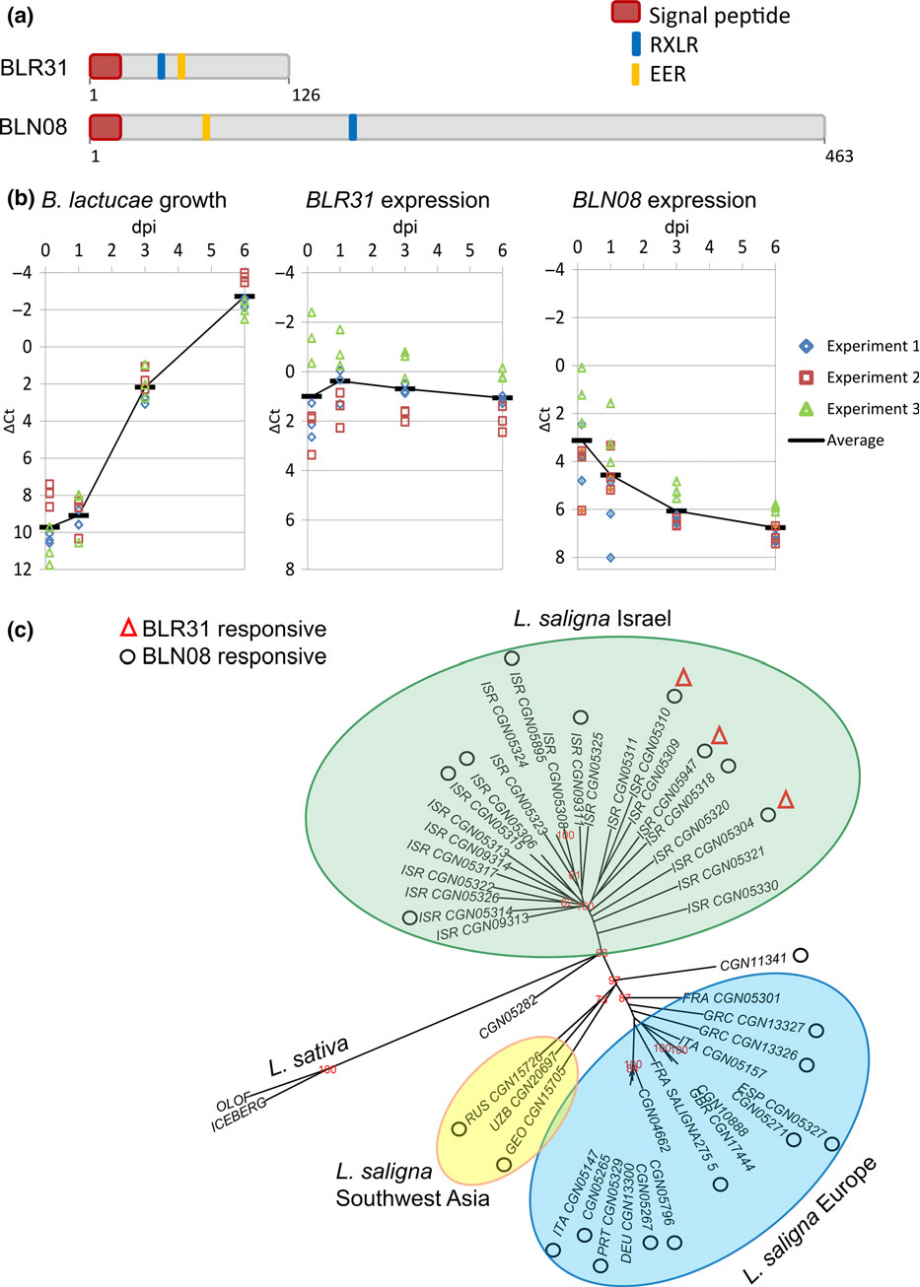
Characteristics of *Bremia lactucae* effectors BLR31 and BLN08, and responsiveness of *Lactuca saligna* accessions. (a) Signal peptide, RXLR motif and EER motif within the amino acid sequences. (b) *Bremia lactucae* Bl:24 growth and candidate effector gene expression during infection of *Lactuca sativa* cv Olof. Growth is inferred by the increase in *Bremia lactucae* actin relative to lettuce actin. Candidate effector gene expression is determined relative to *Bremia lactucae* actin. The *y*‐axis has been reversed to ease interpretation, as lower ∆Ct values indicate higher expression. dpi, days post‐inoculation. (c) Unrooted neighbour‐joining tree constructed of 423 AFLP fragments from 45 *Lactuca saligna* and two *Lactuca sativa* accessions. Three main branches are distinguished: European, Israeli and Southwest Asian. Bootstrap values > 60% (based on 1000 replicates) are indicated in red at the nodes. Triangles, BLR31‐responsive accession; circles, BLN08‐responsive accession.

BLR31 is 126 amino acids long with an N‐terminal signal peptide followed by RLLR and EER motifs that are typical of host‐translocated oomycete effectors (Figs 1a, S1). It shows no homology to proteins in the National Center for Biotechnology Information (NCBI) database. BLN08 (463 amino acids) also has a signal peptide followed by an EER motif, but with an RSLR motif further away. It shows homology (BlastP, 30% identity, E‐value = 1e‐55) to a hypothetical protein (CEG42686.1) of the sunflower downy mildew pathogen (*Plasmopara halstedii*). Expression levels *in planta* were determined by qPCR. Expression of *BLR31* was relatively high, appeared stable over a time course of 6 d, but peaked slightly at 1 dpi. *BLN08* expression was highest at 3 h after infection and decreased markedly during the course of infection (Fig. 1b).

After the first survey, a subset of secondary gene pool accessions was re‐examined for responsiveness to BLN08 and BLR31. Most of the HRs could be confirmed. Only *L. saligna* CGN05310 showed an HR in response to BLN08 in two additional experiments, whereas it did not respond in the germplasm screening (Table S1b).

**Figure 2:**
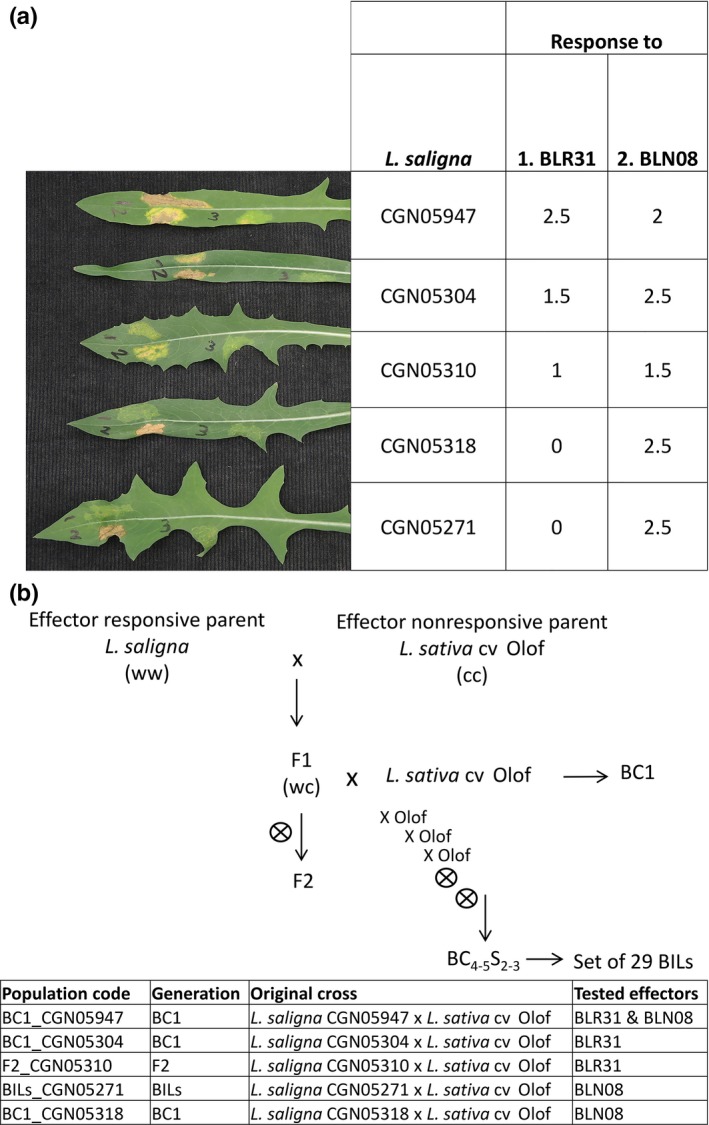
Five BLR31‐ and BLN08‐responsive *Lactuca saligna* accessions and their mapping populations. (a) Plant symptoms (left) after infiltration with BLR31 and BLN08 and their response values (right) in the five selected parental *Lactuca saligna* accessions. Numbers on the leaves indicate infiltrations with (1) BLR31, (2) BLN08 and (3) yellow fluorescent protein (YFP). (b) Crossing scheme to create backcross (BC1), backcross inbred lines (BILs) and F_2_ mapping populations. Allele notation: w, wild lettuce (*Lactuca saligna*) allele; c, cultivated lettuce (*Lactuca sativa*) allele.

In the germplasm screening of 150 lines, BLR31 triggered a consistent HR in three *L. saligna* accessions (CGN05947, CGN05310, CGN05304), all of Israeli origin (Figs 1c, 2a; Table S1b). BLN08 induced a response in 30 of 55 *L. saligna* accessions, as well as in a few *L. sativa*,* L. georgica* and *L. virosa* accessions or lines (Table S1b). BLN08‐responsive *L. saligna* accessions originated from the full geographical range of occurrence of this species and were not restricted to a particular region (Fig. 1c). We focused on responsiveness to BLN08 in *L. saligna* which is a nonhost to *B. lactucae* and is cross‐fertile with *L. sativa*. The species *L. virosa* and *L. georgica* are also highly resistant to *B. lactucae*, but they are less cross‐fertile with *L. sativa*, and most *L. virosa*–*L. sativa* F_1_ hybrids are completely sterile (Lebeda *et al*., 2002; Maisonneuve, 2003).

To determine whether responsiveness to BLR31 and BLN08 co‐segregates with resistance to *B. lactucae*, we tested five *L. saligna × L. sativa* cv Olof mapping populations (BC1, BIL or F_2_) for HR and infection level (Fig. 2). For BLR31, the three Israeli accessions CGN05304, CGN05310 and CGN05947 were selected as responsive parental accessions. For BLN08, we selected as parental accessions: CGN05271, for which a set of BILs was available, CGN05318, and CGN05947. The latter was selected for its response to both BLN08 and BLR31 (Fig. 2a).

### Candidate avirulence effector BLR31 reveals its cognate *R* gene

**Figure 3:**
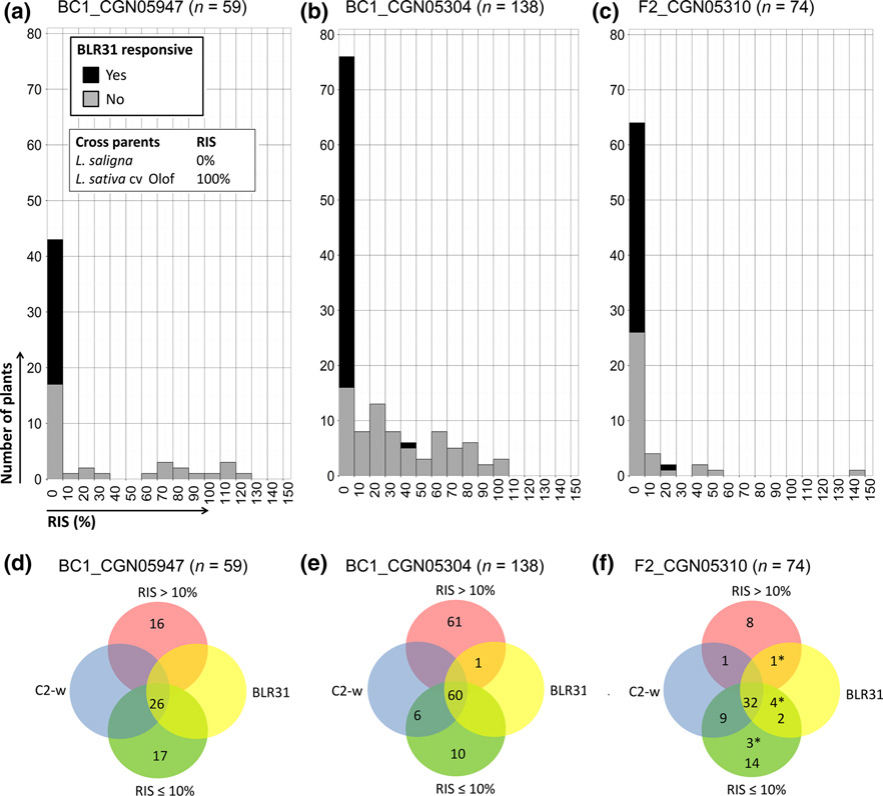
BLR31 responsiveness, Bl:24 infection severity and genotype on the chromosome 2 (C2) mapping locus for segregating populations of the three BLR31‐responsive *Lactuca saligna* parents. (a–c) BLR31‐responsive plants and relative infection severity (RIS) level to race Bl:24 per segregating population. Black bars, plants with a response to BLR31; grey bars, plants without a response to BLR31. Note: all BLR31‐responsive plants in RIS class ≤ 10% did not show any sporulation, and so their exact RIS was 0% (completely resistant). (d–f) Venn diagrams depicting four conditions: BLR31 (yellow), BLR31‐responsive plants; C2‐w (blue), plants with at least one wild lettuce (*Lactuca saligna*) allele on the C2 mapping locus (most closely linked co‐dominant marker depicted); RIS ≤ 10% (green), plants highly resistant to Bl:24; RIS > 10% (red), plants susceptible to Bl:24. *, plants with missing genotype data.

**Figure 4:**
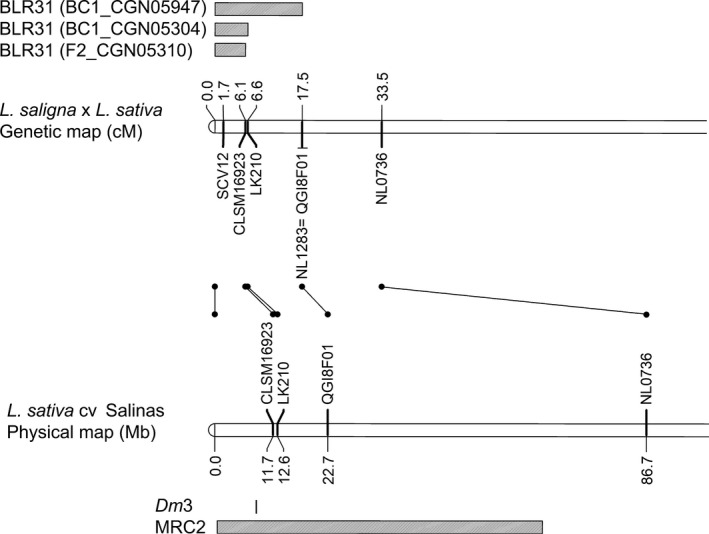
Map interval of responsiveness to BLR31 on chromosome 2 in *Lactuca saligna* CGN05947, CGN05304 and CGN05310. Alignment with the *Lactuca sativa* cv Salinas physical map and MRC2 and *Dm3* intervals is shown below.

Three mapping populations were tested with BLR31, resulting in a range of plant responses, from no reaction to chlorosis and cell death (Table S4). Two groups were distinguished within mapping populations: plants without any response (value 0) and plants with a low to high BLR31 response (the minimum value differed per population). BLR31 responses ranged from 0.8 to 2.5 for BC1_CGN05947, from 0.3 to 1.8 for BC1_CGN05304, and from 0.5 to 2.0 for F2_CGN05310 (Table S4). Responsiveness to BLR31 in all three populations was closely associated with a dominant *L. saligna* allele at the top of chromosome 2 (C2) (Venn diagrams, Fig. 3; Table S4). The map interval overlaps with the *Dm3* region within Major Resistance gene Cluster 2 (MRC2) in *L. sativa* cv Salinas (Christopoulou *et al*., 2015). The smallest genetic interval ranged from 0 to 6.1 cM in *L. saligna* CGN05310 (Fig. 4).

In an adult plant disease test per population, we evaluated for co‐segregation of BLR31 responsiveness with resistance to *B. lactucae* Bl:24, the race from which the candidate effectors were cloned. Control lines behaved as expected, with > 70% RIS for all but one of the susceptible controls, 0–30% RIS for the quantitative resistant controls and 0% RIS for the *L. saligna* accessions (Table S4).

All three populations showed a wide and continuous range of infection severities from 100% (susceptible) to 0% (resistant) RIS (Fig. 3a–c), instead of two discrete classes, namely highly susceptible, similar to the parental *L. sativa* line, and completely resistant, similar to the parental *L. saligna* accession. For each of the three populations, more than half of the plants (73%, 55% and 86%, respectively) were highly resistant (0–10% RIS, Fig. 3a–c). These infection level distributions are indicative of a segregation of quantitative (QTLs) as well as qualitative (monogenic dominant *R* genes) resistance loci.

Responsiveness to BLR31 co‐segregated with complete resistance to Bl:24 in all three populations (Fig. 3; Table S4). All 126 (26 + 61 + 39) BLR31‐responsive plants over three populations were completely resistant with 0% RIS, except for two plants (Fig. 3). One exception in BC1_CGN05304 might be explained by an overestimation of the BLR31‐induced response, as the response to BLR31 was scored at the very low value of 0.3. In F2_CGN05310, the other exception had a relatively low RIS of 25%, suggesting incomplete expression of the BLR31‐associated resistance. In conclusion, the response to BLR31 is mediated by a locus on C2 and associated with complete resistance to Bl:24. Hence, we identified the cognate *R* gene of BLR31 in three *L. saligna* accessions on C2.

### Complexity of stacked resistances

The highly resistant (0–10% RIS) BC1 and F_2_ plants did not all show a response to BLR31 and/or had a *L. saligna* allele at the BLR31 response locus (C2). The resistance in these plants could be explained by other *R* genes, nonhost QTLs or a combination of the two. The percentages of resistant BLR31‐nonresponsive plants in BC1_CGN05947 (52%, 17 of 33 plants, Fig. 3d) and F2_CGN05310 (68%, 17 of 25 plants, Fig. 3f) were close to the 50% and 75% expected for resistant plants in the case of a second *R* gene. Therefore, a dominant *R* gene unlinked to MRC2 was expected for BC1_CGN05947 and F2_CGN05310. To identify these potential additional *R* genes, populations were genotyped with additional genetic markers. All 17 resistant (RIS < 10%) BLR31‐nonresponsive BC1_CGN05947 plants contained a *L. saligna* allele on chromosome 4 (C4) (Fig. S2). The map interval of this *R* gene on C4 in *L. saligna* CGN05947 (119.8–134.8 cM) partly overlaps with MRC4 in *L. sativa* cv Salinas (Fig. S3).

For F2_CGN05310, we could not assign a clear monogenic association to the additional resistance, despite genotyping with 73 markers distributed over the genetic map. Only a weak association with the MRC8(A–C) region on chromosome 8 (C8) was observed. The small number of susceptible (RIS > 10%) plants (*n *=* *10) resulted in too low a resolution for accurate mapping.

In BC1_CGN05304, the percentage of resistant BLR31‐nonresponsive plants was 14% (10 of 71 plants, Fig. 3e), which is not close to the 50% expected resistant plants in the case of one *R* gene. In previous studies, we have shown that the resistance of *L. saligna* CGN05271 to *B. lactucae* is probably based on QTLs and did not show evidence for monogenic dominant resistance loci (Jeuken & Lindhout, 2002; Jeuken *et al*., 2008; Zhang *et al*., 2009a). In six BC1 populations of *L. saligna* accessions × *L. sativa* cv Olof, in which no *R* gene segregated, 4–12% of the plants were highly resistant and explained by nonhost QTLs (A. K. J. Giesbers & M. J. W. Jeuken, unpublished). Therefore, the 14% resistant BLR31‐nonresponsive plants in BC1_CGN05304 can probably be explained by nonhost QTLs. We did not further genotype BC1_CGN05304 as we have no concrete information about the number and locations of QTLs involved.

Summarizing, an additional dominant *R* gene was observed and mapped in *L. saligna* CGN05947 and suggested for *L. saligna* CGN05310. Our results indicate an additional high‐level resistance explained by a combination of NHR QTLs for *L. saligna* CGN05304. This combination of NHR QTLs may also be present in *L. saligna* CGN05947 and CGN05310. However, because of the presence of two *R* genes in relatively small populations, BC1_05947 (*n *=* *59) and F2_05310 (*n *=* *74), the expected number of plants that were resistant because of multiple QTLs only, was very low.

### Responsiveness to BLN08 is not associated with Bl:24 resistance

Responsiveness to BLN08 was tested in progenies of *L. saligna* CGN05947, CGN05271 and CGN05318. First, we screened F_1_ plants from *L. saligna* CGN05947 (*n *=* *4), CGN05271 (*n *=* *2) and CGN05318 (*n *=* *3). A response to BLN08 was absent or minimal in all plants of F1_CGN05947 (average response of 0.2) and present in all plants of F1_CGN05271 and F1_CGN05318 (average response of ≥ 1) (Table S4). The positive result of the latter F_1_s indicates dominance for the responsiveness gene. Second, we screened BC1 populations. BLN08 did not elicit a response in any of the BC1_CGN05947 plants (*n *=* *59).

For *L. saligna* CGN05271, candidate effector BLN08 was infiltrated into 29 BILs, containing one or a few *L. saligna* introgressions in an *L. sativa* cv Olof background, covering *c*. 97% of the *L. saligna* genome. Four plants of a single BIL, preBIL6.2, responded to BLN08, whereas all other BILs were nonresponsive. Genotyping revealed that preBIL6.2 contains a single heterozygous *L. saligna* introgression from 20 to 59 cM on chromosome 8 (C8).

**Figure 5:**
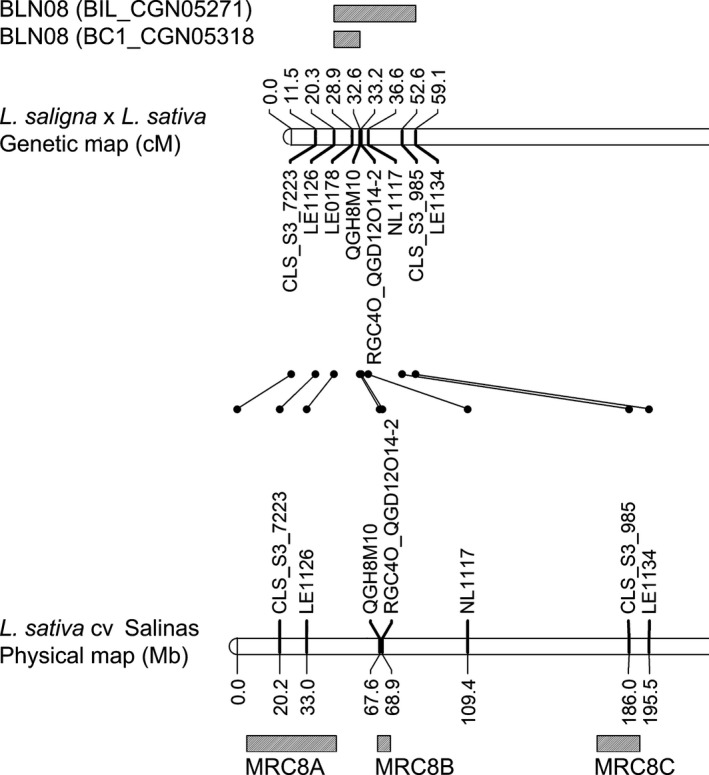
Map interval of responsiveness to BLN08 on C8 in *Lactuca saligna* CGN05318 and CGN05271. Alignment with the *Lactuca sativa* cv Salinas physical map and MRC8(A–C) is shown below.

In BC1_CGN05318, a range of responses to BLN08 from 0.3 to 1.7 was observed in 32 (53%) of 60 plants (Table S4). The response was mapped as a dominant gene on C8 from 20 to 33 cM. For both *L. saligna* accessions CGN05271 and CGN05318, the gene causing responsiveness to BLN08 may overlap with resistance cluster MRC8A or B in *L. sativa* cv Salinas (Fig. 5).

**Figure 6:**
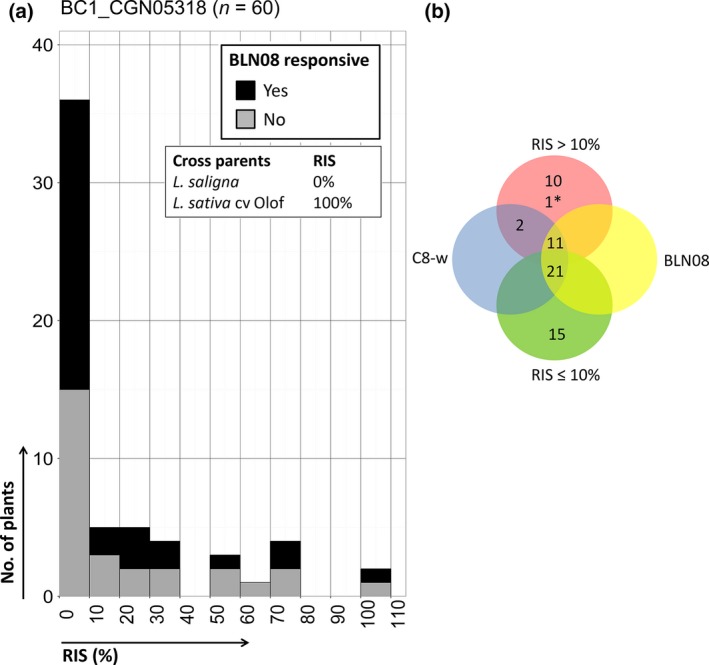
BLN08 responsiveness, Bl:24 infection severity and genotype on the chromosome 8 (C8) mapping locus for the BC1 population of *Lactuca saligna* CGN05318. (a) Response to BLN08 and relative infection severity (RIS) level to race Bl:24. Black bars, plants with a response to BLN08; grey bars, plants without a response to BLN08. (b) Venn diagram depicting four conditions: BLN08 (yellow), BLN08‐responsive plants; C8‐w (blue), plants with one wild lettuce (*Lactuca saligna*) allele on the C8 mapping locus (most closely linked co‐dominant marker depicted); RIS ≤ 10% (green), plants highly resistant to Bl:24; RIS > 10% (red), plants susceptible to Bl:24. *, plants with missing genotype data.

BLN08 responsiveness did not co‐segregate with *B. lactucae* Bl:24 resistance in adult disease tests in *L. saligna* CGN05271‐ and CGN05318‐derived progeny. Control lines behaved as expected, with > 70% RIS for the susceptible controls, 0–30% RIS for the quantitative resistant controls, and 0% RIS for the *L. saligna* accession (Table S4). BLN08‐responsive preBIL6.2 plants (*n *=* *2) of *L. saligna* CGN05271 were as susceptible to Bl:24 as *L. sativa* cv Olof (susceptible parent). The response to BLN08 occurred in all infection severity classes (0–100%) of BC1_CGN05318 (Fig. 6). However, 60% (36 of 60) of the BC1_CGN05318 plants were highly resistant (RIS ≤ 10%, Fig. 6), indicating the presence of a dominant *R* gene (50% expected under Mendelian segregation), possibly with the remaining 10% of resistant plants explained by QTLs for NHR. The other 40% of plants showed a wide range of infection severities (Fig. 6), indeed indicating segregation for QTLs. Genetic mapping showed that a dominant *R* gene explained 83% (30 of 36, Table S4) of the resistant plants. This gene mapped on C1 between 32–73 cM, which overlaps with MRC1 in *L. sativa* cv Salinas (Fig. S4). Six (17%) resistant plants without a *L. saligna* allele at the C1 map interval are probably explained by a combination of nonhost QTLs.

Taken together, the BLN08‐induced response in *L. saligna* CGN05947 was, for unknown reasons, completely absent in its F_1_ and BC1 progeny, whereas it was mapped to C8 in *L. saligna* CGN05271 and CGN05318. In *L. saligna* CGN05271‐ and CGN05318‐derived progeny, the response to BLN08 was not associated with Bl:24 resistance. Instead, an *R* gene on C1 that was not associated with BLN08 responsiveness was identified in *L. saligna* CGN05318.

### Intensity differences in plant responses to effectors

Lowest and highest responses to BLR31 and BLN08 of *L. saligna* and its derived progenies varied from light chlorosis (0.5) to strong cell death (2.5) (Fig. S5). We were interested in whether these intensity differences of effector‐induced responses reflect *L. saligna* allele dose differences. Therefore, we compared the values of plants with one allele (F_1_ and responsive BC1/F_2_) and two alleles (responsive F_2_ and parental *L. saligna* accessions) per accession (Fig. S5). Average responses to effectors were significantly higher (*P *<* *0.001) for plants with two *L. saligna* alleles compared with plants with one *L. saligna* allele in all accessions, except for CGN05310 (*P *>* *0.1) (Fig. S5). However, the response ranges of plants within both the one allele and the two allele dose groups were wide and overlapping (except for CGN05318, which were wide but not overlapping). Therefore, individual plants cannot be classified for the responsive allele dose based on their effector response value alone (except for CGN05318, Fig. S5).

Overall, our BLR31 and BLN08 data revealed that resistance in the tested *L. saligna* accessions is composed of diverse complexities of stacked resistances: qualitative resistance by *R* genes and quantitative resistance by nonhost QTLs. The induced response to BLR31 in a narrow range of accessions was associated with Bl:24 resistance, whereas the response to BLN08 in a broad range of accessions was not.

## Discussion

Our first objective was to obtain a broader picture of responsiveness to effectors in the nonhost *L. saligna*. Second, we wanted to verify whether effector‐induced responses in a nonhost are associated with disease resistance to the effector‐producing pathogen strain. Unlike all other effector screenings in nonhost species, our study verified the co‐segregation of effector‐induced responsiveness with resistance in segregating populations. This analysis of co‐segregation was possible because of the exceptional situation in which the nonhost, *L. saligna*, is cross‐fertile with the host species, cultivated lettuce. We detected a response to BLR31 in 5% (3 of 55) of the *L. saligna* accessions, and a response to BLN08 in 55% (30 of 55) of the *L. saligna* accessions.

BLR31‐induced responsiveness co‐segregated with complete resistance to *B. lactucae* Bl:24. Therefore, BLR31 is a candidate avirulence effector of lettuce downy mildew, and probably interacts with an *R* gene on C2 (in *L. saligna* CGN05304, CGN05947, CGN05310). This resistance locus is new for the species *L. saligna*. All other *R* genes against *B. lactucae* that have been mapped in *L. saligna* are located on C1 (*n *=* *3) and C9 (*n *=* *1) (Parra *et al*., 2016).The *R* gene on C2 co‐localized with MRC2 on C2 of *L. sativa*. We are unaware of the presence of MRCs in *L. saligna* because of the absence of a genome sequence, but similar MRCs in *L. sativa* and *L. saligna* are conceivable as a result of synteny. In *L. sativa*, MRC2 contains 61 NLR‐encoding genes (Christopoulou *et al*., 2015) and nine known *Dm* genes, of which *Dm3* is cloned and explained by an NLR gene (Parra *et al*., 2016). This indicates that the *L. saligna* C2 *R* gene may be a putative NLR gene. *R* gene cloning could functionally prove this hypothesis. Effector intensity differences of BLR31‐responsive plants further showed that an HR can vary from chlorosis to cell death, as both phenotypes co‐segregated with one allele of the *R* gene.

BLN08 induced a response in the majority of tested *L. saligna* accessions. Interestingly, the response to BLN08 did not co‐segregate with Bl:24 resistance. However, the response to BLN08 was mapped to C8 and co‐localized with MRC8(A–B) on C8 in *L. sativa*. MRC8A and MRC8B contain 42 NLR‐encoding genes (Christopoulou *et al*., 2015) without known *Dm* genes (Parra *et al*., 2016). Previously, the induced response to BLG01 in a broad range of *L. saligna* accessions (in 47 of 52 accessions; 90%) was also not associated with disease resistance. The response to BLG01 in *L. saligna* CGN05271 localized to the bottom of C9 (Stassen *et al*., 2013), which is now known to co‐localize with MRC9C in *L. sativa* (Christopoulou *et al*., 2015). The fact that induced responses to two candidate effectors in a broad range of nonhost *L. saligna* accessions do not co‐segregate with resistance, whereas the induced response to BLR31 in a narrow range of accessions does, raises the question of whether this is a pattern. Are responses to effectors induced in a narrow range of accessions directly associated with resistance, whereas responses induced in a broad range of accessions are not (directly) associated with resistance? As a result of the currently limited number of *B. lactucae‐*derived candidate effectors that induce an HR in *L. saligna* (*n *=* *3), we cannot yet answer this question.

A potential explanation for the lack of co‐segregation between responsiveness to an effector and resistance is that the *R* gene is not functional against the *B. lactucae* isolate from which the effector has been cloned, because other effectors in this isolate suppress the resistance response (King *et al*., 2014; Teper *et al*., 2014). It is possible that the *R* gene is effective against older downy mildew isolates, in which the *R* gene‐suppressing effectors have not yet evolved. Another possibility is that a second unlinked gene is required for resistance (Cooley *et al*., 2000; Kachroo *et al*., 2000). Or, a functional version of a second tightly linked gene, as in NLR pairs (Cesari *et al*., 2014; Williams *et al*., 2014), may be required as an inducer of disease resistance signalling. The *R* gene might also confer resistance against another pathogen and recognize the downy mildew effector as a side effect. Other explanations for the absence of co‐segregation between responsiveness to an effector and disease resistance may be a lack of proper effector translocation in the host, mis‐timed expression of the effector or the *R* gene, or incomplete resistance mediated by the *R* gene (Krasileva *et al*., 2011; Goritschnig *et al*., 2012). The latter explanation can be excluded, as the average resistance level of BLN08‐responsive BC1 plants was not significantly lower than that of BLN08‐nonresponsive BC1 plants.

Although *L. saligna* CGN05947 showed a clear BLN08‐induced cell death response, its derived F_1_ and BC1 plants did not respond to BLN08. Possibly, *L. sativa* alleles (50% per F_1_ plant and, on average, 75% per BC1 plant) interfere with BLN08‐induced cell death in the hybrid plants. Variation at the BLN08 response locus, like unexpected heterozygosity, in the *L. saligna* parent can be excluded, because *L. saligna* CGN05947 (*n *=* *26) showed a consistent response to BLN08 in three independent experiments. These deviating responses between F_1_ hybrid plants of the three BLN08‐tested *L. saligna* accessions make us wonder how F_1_ plants of the other 27 responsive *L. saligna* accessions will respond. The testing of more of these F_1_s (and their BC1s) would provide a better reflection of the true character of the BLN08 response and could be tested in future experiments, but requires very laborious and difficult crossing work.

The recognition of effectors in a nonhost is often hypothesized to contribute to NHR (Wroblewski *et al*., 2009; Schulze‐Lefert & Panstruga, 2011; Lee *et al*., 2014; Adlung *et al*., 2016). However, HRs are also histologically observed in susceptible lettuce and potato cultivars, and are associated with both host and NHR in potato (Vleeshouwers *et al*., 2000; Zhang *et al*., 2009b). Therefore, the presence of responsiveness to an effector in a nonhost does not necessarily mean that this contributes to NHR. Our current and previous results characterized nonhost responses to three candidate effectors. They showed that the responses to BLN08 and BLG01 in *L. saligna* do not co‐segregate with disease resistance, although they segregate as single dominant loci and co‐localize with NLR loci, as has been found previously for *Pseudomonas* effectors in lettuce (Wroblewski *et al*., 2009).

In addition, in host–pathogen interactions, an effector‐induced cell death response does not necessarily imply a functional *R* gene mounting an HR to the pathogen delivering that effector. For instance, recognition of *Hyaloperonospora arabidopsidis* (*Hpa*) effectors in Arabidopsis did not inhibit growth of *Hpa* strains expressing the effector (Krasileva *et al*., 2011; Goritschnig *et al*., 2012).

A complexity of multiple and diverse resistances was identified in the *L. saligna* accessions. In addition to the resistance explained by the BLR31‐responsive *R* gene, additional highly resistant hybrid plants without a response to BLR31 were observed in all three mapping populations tested with BLR31. Also, in BC1_CGN05318 tested with BLN08, highly resistant plants without a response to BLN08 were observed. Two dominant monogenic *R* genes (on C4 for CGN05947 and C1 for CGN05318) and some evidence for a putative *R* gene on C8 for CGN05310 explained all or a large part of the highly resistant plants in three populations. For the remaining highly resistant BC1 plants (6% of total), no evidence for dominant monogenic resistance was found. Based on our previous genetic studies in *L. saligna*, we assume that these highly resistant plants are explained by several quantitative effect *R* genes (nonhost QTLs), as discovered in *L. saligna* CGN05271 (Jeuken & Lindhout, 2002; Jeuken *et al*., 2008; Zhang *et al*., 2009a). These resistant plants explained solely by nonhost QTLs suggest that NHR is independent of *R* genes.

The general frequency and distribution of *R* genes in the species *L. saligna* is unknown, although a few *R* genes have been found in *L. saligna* accessions and those with known genetic positions co‐localize with *L. sativa* MRC1 and MRC9C (Parra *et al*., 2016). Our results add three new *R* genes and one putative *R* gene, at one known (MRC1) and three new resistance loci for *L. saligna* (co‐localization with MRC2, MRC4 and MRC8A‐B in *L. sativa*, respectively). These findings suggest that the presence of *R* genes in *L. saligna* accessions is not uncommon (at least two *R* genes out of five *L. saligna* accessions from a nontargeted approach). This may be surprising, as *R* genes are typically assumed to have evolved under selection pressure by a harmful pathogen. As *L. saligna* is a nonhost to *B. lactucae*, it remains an interesting question as to what selective forces have resulted in the evolution of so many *R* genes. Possibly, *L. saligna* has previously been a host for *B. lactucae* or for an extinct related pathogen, and the *R* genes are remnants of this ancient host status.

We have shown that *R* gene discovery by effectors is successful if responsiveness of the plant is associated with resistance against the pathogen (as for BLR31). However, it leads to the wrong track if there is no association (as for BLN08 and BLG01). Furthermore, *R* gene discovery in *L. saligna* may very well be possible without the use of effectors, as *R* genes appear to be fairly common in *L. saligna*.

In summary, we have identified a candidate avirulence effector of *B. lactucae* (BLR31) and its cognate *R* gene in *L. saligna*. *Lactuca saligna* accessions contain QTLs for NHR and some also carry one or more *R* genes. The presence of resistant hybrid plants with solely nonhost QTLs and no *R* genes against Bl:24 suggests that *R* genes are not required for NHR, although they might still contribute. The broadly induced response to BLN08 was not associated with resistance, similar to our earlier finding with the broadly induced response to BLG01 (Stassen *et al*., 2013). Therefore, effector‐induced responses in a nonhost may be independent of the NHR mechanism.

## Author contributions

A.K.J.G. and M.J.W.J. designed, performed and analysed the experiments, with the contribution of A.J.E.P. and G.V.d.A. for candidate effector identification and expression analysis. A.K.J.G. and M.J.W.J. wrote the manuscript. R.G.F.V., R.E.N. and all other authors were involved in revising the manuscript critically.

## Supporting information

Please note: Wiley Blackwell are not responsible for the content or functionality of any Supporting Information supplied by the authors. Any queries (other than missing material) should be directed to the *New Phytologist* Central Office.

**Fig. S1** Nucleotide and amino acid sequence of BLR31 and BLN08.
**Fig. S2** Histogram of the BC1 population of *Lactuca saligna* CGN05947, showing BLR31 responsiveness and the *R* gene on C4.
**Fig. S3** Map interval of the *R* gene on C4 in *Lactuca saligna* CGN05947.
**Fig. S4** Map interval of the *R* gene on C1 in *Lactuca saligna* CGN05318.
**Fig. S5** Range of hypersensitive response (HR) scores for effector‐responsive plants per *Lactuca saligna* accession.

**Table S1** Germplasm set and candidate effector screening results
**Table S2** List of primers used in this study
**Table S3** Chromosome numbering and orientation
**Table S4** Effector responsiveness, relative infection severity (RIS) and genetic marker data per segregating population
