# Supplementary material for: Effector‐mediated discovery of a novel resistance gene against Bremia lactucae in a nonhost lettuce species
Source: New Phytol. 2017 Aug 21;216(3):915–26. doi: 10.1111/nph.14741 (PMC5656935; doi:10.1111/nph.14741)
Supplement: Supplementary file 1 — Fig. S1 Nucleotide and amino acid sequence of BLR31 and BLN08. Fig. S2 Histogram of the BC1 population of Lactuca saligna CGN05947, showing BLR31 responsiveness and the R gene on C4. Fig. S3 Map interval of the R gene on C4 in Lactuca saligna CGN05947. Fig. S4 Map interval of the R gene on C1 in Lactuca saligna CGN05318. Fig. S5 Range of hypersensitive response (HR) scores for effector‐responsive plants per Lactuca saligna accession. [file NPH-216-915-s001.pdf]

New Phytologist Supporting Information Figures S1-S5

Article title: Effector-mediated discovery of a novel resistance gene against *Bremia lactucae* in a nonhost lettuce species

Authors: Anne K.J. Giesbers, Alexandra J.E. Pelgrom, Richard G.F. Visser, Rients E. Niks, Guido Van den Ackerveken and Marieke J.W. Jeuken

Article acceptance date: 26 June 2017

>BLR31

```
   1 ATGCTTCTTT CCCGTGCCAT CTCTGTACTC GCCCTACTCG CATGTATTCG TTGTGGGGTG
  61 CACGCACAAA ACACGGAACA AAATCTTAAG ACTCAACTAA CCACCGACAG CGCGATGATC
 121 ACTTCGCAGC GGCTTCTCAG AACCAGCGTG GACTTTAAAG ACAGTGAAGA ACGTTGGCCT
 181 ACTGAAAGTA GTAGGATCAG AAGTGCCATT AAGGACTATT TCCGTGAATT TCCAGAAAAA
 241 GTAAGTATTG CTATGGCGAT AAGACAGATA GATGCACACG GCGTTCGTCA TGTTGAGAAG
 301 GTGCTCTCAC AATACAAATT CCCTGCCGCA GACCAAGGAA ACATACGATT AGCGATAATT
 361 CATCACAAAG CTCCAAAATA A

   1 MLLSRAISVL ALLACIRCGV HAQNTEQNLK TQLTTDSAMI TSQRLLRTSV DFKDSEERWP
  61 TESSRIRSAI KDYFREFPEK VSIAMAIRQI DAHGVRHVEK VLSQYKFPAA DQGNIRLAII
 121 HHKAPK
```

>BLN08

```
   1 ATGAATTTGC ACTTCTTGCT ACTGTCCCTA CTCCTTACAA CAGCGAATGC CGCGCTTAAT
  61 AAGAATGCAA GCGAAAATAG CGCTCAACCC CCCTCAAGCC TCCCCGAACA TAGCCGTCCC
 121 TTCCTTCCTA AAAGTCCGAA CAAAGACCTC GCAATCCAAG GCCCTCGATT CAACGATGCA
 181 CATGACATGA CTGGCTCTCA AGCCACATCA ATTGAAGAAA GAAACCTTCT GGACTCGATT
 241 AAGCACACAA CGCTCGATGC CGTCTACAAA TTGGCCGCGA AACTCCGTGC GAGTCCGCGA
 301 TTTATGTTTT ATGCCATGGG AATTTTCATT TCGCGCCTTA CGAAAAAGTT GCACAAAAAA
 361 ATCAATCTCT ACCAGTGGCT TCTTTACGTA GACAAGCACA TGTTTCAACC GTGCAAGTCT
 421 CACGACGAGC TCTTGGCCGC AAGCAGCACT TTCTTTTCCT TCTTTCAACG AAAATTTACC
 481 GACGTACAGC TTGCGGGGTT CTTTCGATCG CTTCGAACAT ATCCGGGTCT GTCAAACTTG
 541 GGAGATTGGA TGCAAACGTA CATGGCTACA AACGTCGCTA CGAGCTCAGC GATGCGGGAG
 601 GCGTGGAGTT GGTATGGCGA TACGATCGAC GTTGTTTTCA AGACCCTGCG CGTTGAAAAC
 661 GAAGCAGATC TTGTCGGTAG CCGTGTGGTG ACTGCGTGGT TAGAATACTG TCATGCGCGC
 721 CGTCACGTGG CCACGCGAGA TCCAATGATT AATCTCATAG CGCTAGAAAA CATCGTGCGG
 781 TTGCTCAAGA CGACCAAGCC GGATCAAGAC CTAAAGACCG TATTTAAGAC GTTTAGTGGT
 841 GTAAATGGGA TGGAAGAATT TGCCAAGGAA TTGATTGAAG TCATTGAGCG GGAAGCACAA
 901 ATTGAAGCCT GGGCGGCGAA GAAGGTGCAT CCATCGAAAG TCTATGACGA GTTGGAGCTA
 961 GGTACGACGA ATTCGATTGA TATTACCCGA TTTATTCAGT GGCTTCGATA TTTGCAAAAG
1021 ATTCAAGTCG AAAACGATGT TTTTGTGCAT TTTTCTAAAA CGATTCCTAA AGGACAAGAG
1081 ATTGAGTATG CATCGATTTT AAAAGACATG ACACTCTTTC CGGATTTAGA AACGTTCTCG
1141 AAAGACCTAC GTAGCGTCTT GTATAAAAAC TGGGCCGCCG ACACCGACAT GACGCCGCTT
1201 ATGCTGATGA AGCGCATGAC TTCTTCCGTT GCTACTCTCT CGAGTATTGA TCCAAAACGC
1261 GTGGTGCTGC TTGAGTACAC CAAGTATTTT ATCATTCGAT ATAACGCGGC TTTATGGCCA
1321 CAATTCCAAA AAATAGTGGA AAAGAATGGT ATTGTTGCTG CGGTGAAATT TGCATCGAAT
1381 GTAAACCTTT GA

   1 MNLHFLLLSL LLTTANAALN KNASENSAQP PSSLPEHSRP FLPKSPNKDL AIQGPRFNDA
  61 HDMTGSQATS IEERNLLDSI KHTTLDAVYK LAAKLRASPR FMFYAMGIFI SRLTKKLHKK
 121 INLYQWLLYV DKHMFQPCKS HDELLAASST FFSFFQRKFT DVQLAGFFRS LRTYPGLSNL
 181 GDWMQTYMAT NVATSSAMRE AWSWYGDTID VVFKTLRVEN EADLVGSRVV TAWLEYCHAR
 241 RHVATRDPMI NLIALENIVR LLKTTKPDQD LKTVFKTFSG VNGMEEFAKE LIEVIEREAQ
 301 IEAWAAKKVH PSKVYDELEL GTTNSIDITR FIQWLRYLQK IQVENDVFVH FSKTIPKGQE
 361 IEYASILKDM TLFPDLETFS KDLRSVLYKN WAADTDMTPL MLMKRMTSSV ATLSSIDPKR
 421 VVLLEYTKYF IIRYNAALWP QFQKIVEKNG IVAAVKFASN VNL
```

Fig. S1 Nucleotide and amino acid sequence of candidate effector BLR31 and BLN08. Red: signal peptide, blue: RXLR motif, yellow: EER motif.

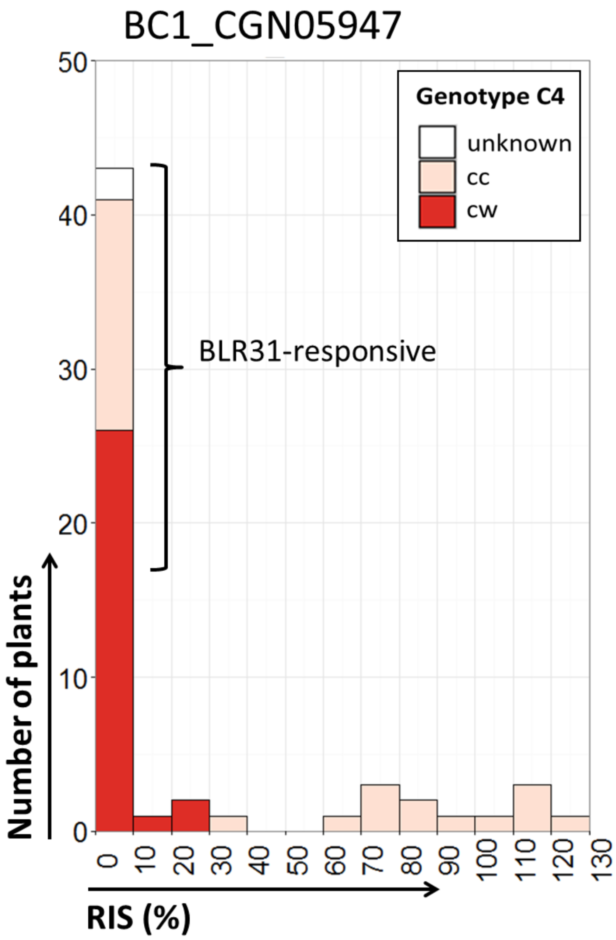

Fig. S2 Distribution of segregating population BC1_CGN05947 for genotype on the chromosome 4 (C4) *R* gene locus, BLR31 responsiveness, and relative infection severity (RIS) to *Bremia lactucae* race Bl:24*.* Red: plants with a *Lactuca saligna* allele on the C4 locus (cw), pink: plants without a *Lactuca saligna* allele on the C4 locus (cc), white: genotype unknown.

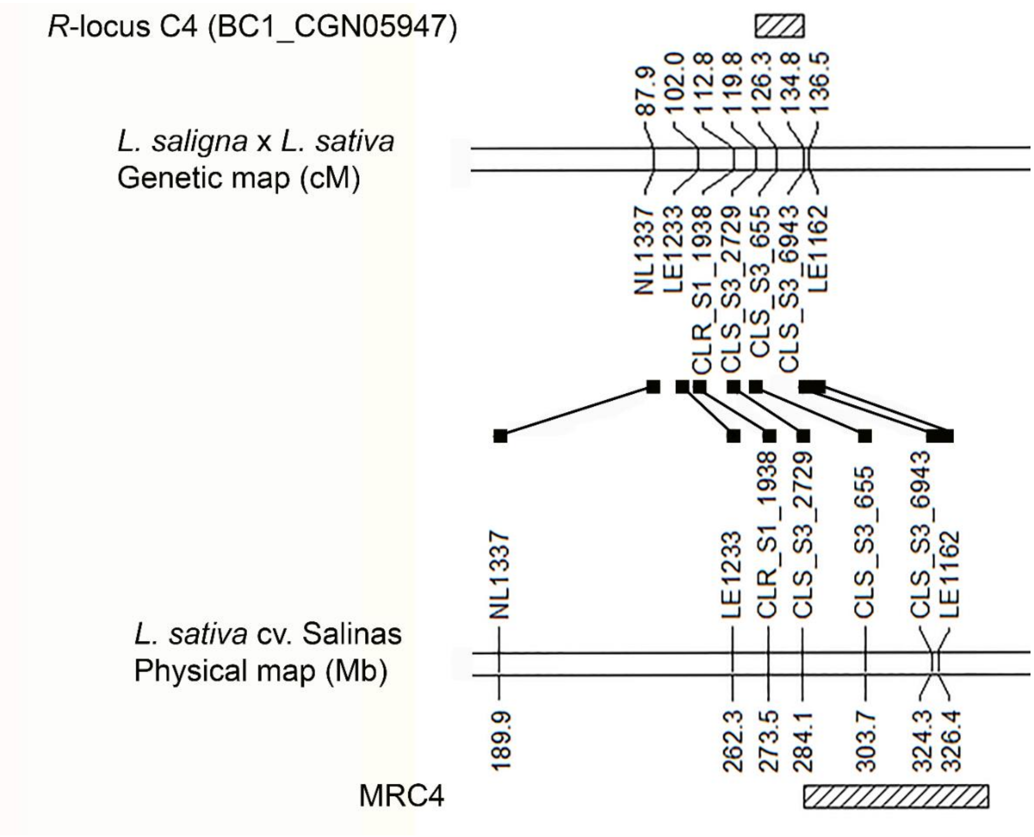

Fig. S3 Map interval of the *R* gene on chromosome 4 (C4) in *Lactuca saligna* CGN05947. Major resistance cluster 4 (MRC4) in *Lactuca sativa* cv. Salinas is shown below the physical map. The genetic map is derived from an F2 population of *Lactuca saligna* CGN05271 x *Lactuca sativa* cv. Olof. The physical map is from *Lactuca sativa* cv. Salinas (http://lgr.genomecenter.ucdavis.edu/).

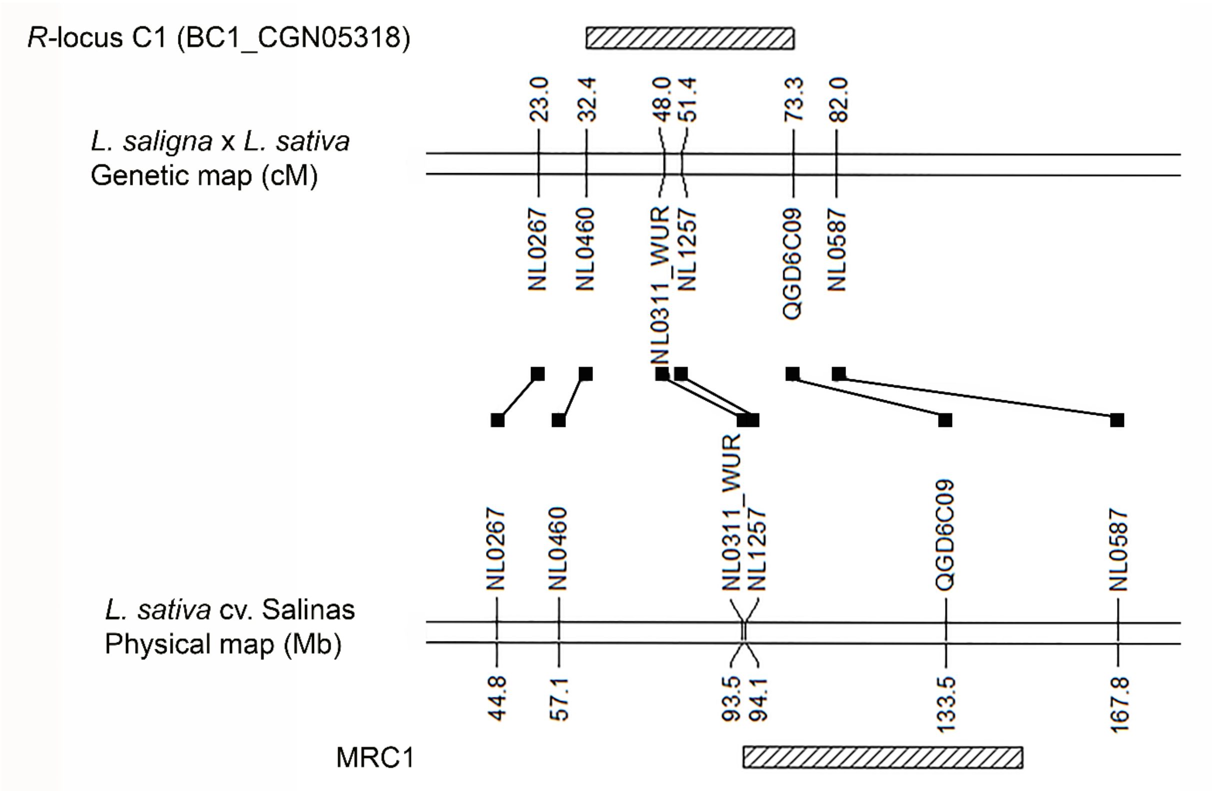

Fig. S4 Map interval of the *R* gene on chromosome 1 (C1)) in *Lactuca saligna* CGN05318. Major resistance cluster 1 (MRC1) in *Lactuca sativa* cv. Salinas is shown below the physical map. The genetic map is derived from an F2 population of *Lactuca saligna* CGN05271 x *Lactuca sativa* cv. Olof. The physical map is from *Lactuca sativa* cv. Salinas (http://lgr.genomecenter.ucdavis.edu/).

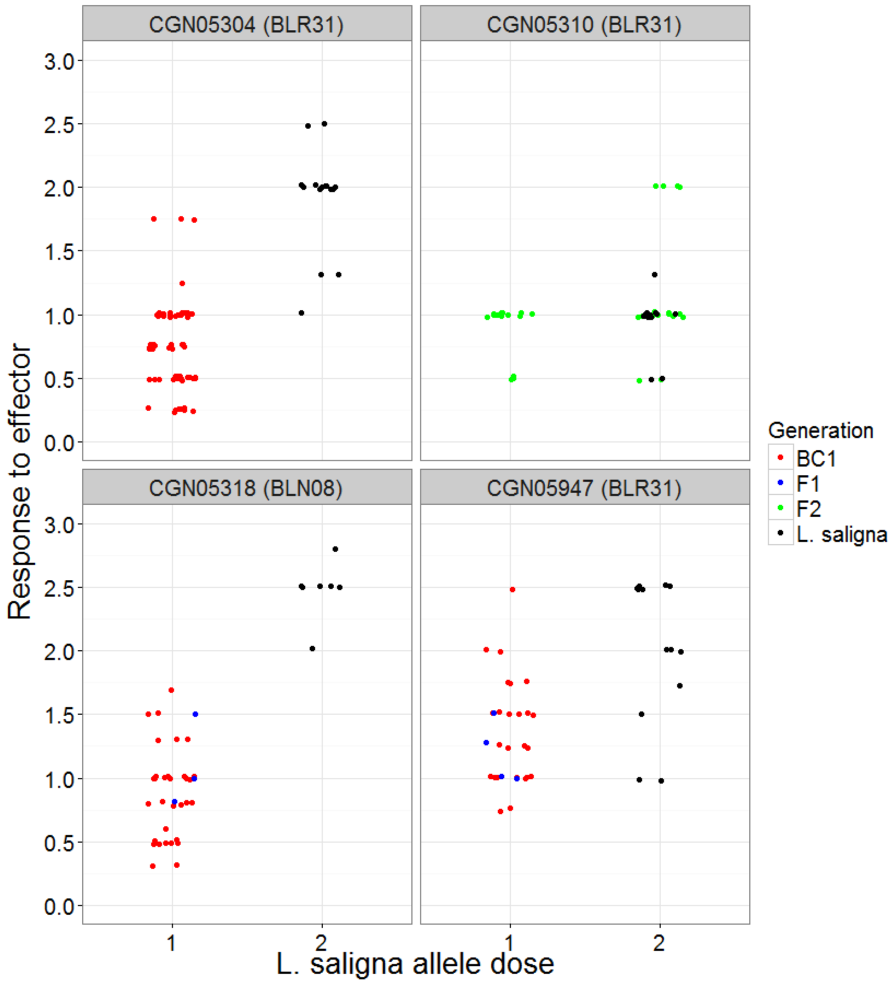

	Allele dose 1		Allele dose 2		Allele dose 1 vs 2
**Accession**	**Mean**	**SD**	**Mean**	**SD**	**p-value (Anova)**
**CGN05304**	0.8	0.4	1.9	0.4	<0.001
**CGN05310**	0.9	0.2	1.1	0.4	0.123
**CGN05318**	0.9	0.4	2.5	0.2	<0.001
**CGN05947**	1.4	0.4	2.1	0.6	<0.001

Fig. S5 Range of effector scores for responsive plants with one *Lactuca saligna* allele (BC1, F1, F2) and plants with two *Lactuca saligna* alleles (F2, *L. saligna*) per accession. A jitter function was used to add a small amount of noise, in order to make all data points visible.
